# A monocentric phase I study of vemurafenib plus cobimetinib plus PEG-interferon (VEMUPLINT) in advanced melanoma patients harboring the V600*BRAF* mutation

**DOI:** 10.1186/s12967-020-02680-7

**Published:** 2021-01-06

**Authors:** Ester Simeone, Giosuè Scognamiglio, Mariaelena Capone, Diana Giannarelli, Antonio M. Grimaldi, Domenico Mallardo, Gabriele Madonna, Marcello Curvietto, Assunta Esposito, Fabio Sandomenico, Francesco Sabbatino, Nicholas L. Bayless, Sarah Warren, SuFey Ong, Gerardo Botti, Keith T. Flaherty, Soldano Ferrone, Paolo A. Ascierto

**Affiliations:** 1 Istituto Nazionale Tumori–IRCCS–Fondazione G Pascale, Naples, Italy; 2 Istituto Nazionale Tumori Regina Elena, IRCCS, Rome, Italy; 3 Oncology Unit, AOU San Giovanni Di Dio E Ruggi D’Aragona, Salerno, Italy; 4 Parker Institute for Cancer Immunotherapy, San Francisco, CA USA; 5 NanoString Technologies, Seattle, WA USA; 6 Massachusetts General Hospital Cancer Center, Boston, MA USA

**Keywords:** Malignant melanoma, BRAF inhibitor, MAP kinase, Interferon

## Abstract

**Background:**

Studies carried out in vitro and in a mouse model have shown that BRAF inhibitors enhance the effects of IFN-α on *BRAF*V600E melanoma cells through the inhibition of ERK. Therefore, the combination of vemurafenib and IFN-α in patients with *BRAF*V600E melanoma may provide therapeutic benefits; MEK inhibition may prevent the reactivation of the MAPK pathway induced by BRAF inhibitor resistance.

**Patients and methods:**

In a phase I study, adult patients with advanced *BRAF*V600-mutated melanoma were treated with vemurafenib + PEG-IFN-α-2b or vemurafenib + cobimetinib + PEG-IFN-α-2b, to assess the safety of the combination and the upregulation of IFN-α/β receptor-1 (IFNAR1).

**Results:**

Eight patients were treated; 59 adverse events with four serious ones (three related to study treatments) were reported. Patients with a pre-treatment IFNAR1 expression on ≤ 35% melanoma cells had a median progression-free survival of 12.0 months (range: 5.6–18.4 months) and a median overall survival of 31.0 months (range: 19.8–42.2 months), while patients with a pre-treatment IFNAR1 expression on > 35% of melanoma cells had a median progression-free survival of 4.0 months (range: 0–8.8; p = 0.03), and a median overall survival of 5 months (p = 0.02). Following treatment, responders had higher levels of growth-suppressor genes, including *GAS1* and *DUSP1*, and genes involved in a metabolically robust immune response, including *FAP*.

**Conclusion:**

Our study supports the overall safety of the vemurafenib + PEG-IFN-α-2b + cobimetinib combination. IFNAR1 expression levels correlated with response to treatment, including survival. Vemurafenib + PEG-IFN-α-2b + cobimetinib would have difficulty finding a niche in the current treatment scenario for advanced melanoma, but we speculate that our findings may contribute to identify subjects particularly responsive to treatment.

*Trial registration:* The study was registered at clinicaltrials.gov (NCT01959633). Registered 10 October 2013, https://clinicaltrials.gov/ct2/show/NCT01959633

## Introduction

Malignant melanoma accounts for most deaths due to skin cancer. Although public campaigns advocating early detection have led to significant reductions in mortality rates and combined 5-year relative survival rates for all stages are calculated to be > 90%, incidence rates continue to increase [1, 2]. In recent years, immunotherapy and targeted therapies have changed the treatment scenario for advanced melanoma [3, 4]. In particular, targeted therapy with BRAF–MEK inhibitors provides a long-lasting benefit [5, 6]. Nevertheless, melanoma cells adapt to the blocking of BRAF and MEK, becoming able to thrive even under pharmacological pressure [7].

Approximately 50% of human melanomas are driven by *BRAF* mutations, characterized by aggressive growth and a highly immunosuppressive tumor microenvironment [8]. Mutant *BRAF*V600 leads to the activation of the MAP kinase (MAPK) pathway [9], with a sustained proliferation and survival of melanoma cells and downregulation of molecules, such as HLA class I antigens and tumor antigens, which mediate interactions of melanoma cells with immune cells [10–15]. MAPK pathway activation is also known to downregulate the expression of type I IFN-α receptor-1 (IFNAR1), which mediates the effects of IFN-α, a cytokine used for the adjuvant treatment of high-risk melanoma [16–19]. Specifically, ERK activation upregulates βTrcp2/HOS protein, a E3 ubiquitin ligase that increases the ubiquitination and degradation of IFNAR1 [20, 21]. As a result, IFNAR1 levels and its signaling are downregulated. We, and others, have shown that BRAF inhibitors (BRAF-I) enhance the antiproliferative and immunomodulatory effects of IFN-α on *BRAF*V600E melanoma cells because the inhibition of ERK activation by BRAF-I upregulates IFNAR1 expression on melanoma cells [15]. These results argue in favor of the possibility that the combination of the BRAF-I vemurafenib and IFN-α may represent a useful combinatorial strategy for the treatment of patients with *BRAF*V600 melanoma. However, reactivation of the MAPK pathway caused by BRAF-I resistance is expected to reactivate ERK activity, which, in turn, would downregulate IFNAR1 expression on melanoma cells. MEK inhibitor (MEK-I) administration would inhibit the reactivation of the MAPK pathway induced by BRAF-I resistance and, as a result, would restore IFNAR1 expression on melanoma cells when it is downregulated by ERK reactivation [22]. These findings have provided a strong rationale to test the therapeutic efficacy of the BRAF-I + MEK-I + IFN-α combination for the treatment of *BRAF*V600E melanoma patients. In a phase I study, we have assessed the safety of this combination and its ability to upregulate the IFN-α signature-related gene-expression levels in patients with *BRAF*-mutated advanced melanoma.

## Patients and methods

### Design

An open-label, single-arm, dose-escalation, monocenter, phase I study was conducted at Istituto Nazionale Tumori IRCCS – Fondazione “G Pascale” in Naples (Italy). The study was performed in accordance with the current version of the declaration of Helsinki (52nd WMA General Assembly, Edinburgh, Scotland, October 2000), in agreement with the International Conference on Harmonisation guidelines on Good Clinical Practice, in compliance with the requirements of Italian Istituto Superiore di Sanità and Agenzia Italiana del Farmaco. The study gained full approval from the Ethical Committee of the Istituto Nazionale Tumori IRCCS–Fondazione “G Pascale” on 15 January 2014. All patients have provided written informed consent to participate in the study prior to being screened. The study was registered at clinicaltrials.gov (NCT01959633).

### Patients

Adult patients with advanced melanoma were eligible provided that they met the following criteria: untreated or pretreated (no more than one treatment) metastatic melanoma at unresectable stage IIIb–IV, histologically confirmed diagnosis, showing *BRAF*V600 mutations; measurable disease by RECIST v 1.1; Eastern Cooperative Oncology Group (ECOG) performance status (PS) 0–1; successfully recovered from all the secondary side-effects of the previous systemic therapy; appropriate hematologic, hepatic and renal functionality assessed within 7 days preceding the start of therapy; a negative pregnancy test performed within 7 days before beginning the therapy for premenopausal women; effective contraception during therapy and for at least 6 months after the treatment. Exclusion criteria were: prior systemic treatment with BRAF-I or MEK-I, or IFN-α; symptomatic brain metastases; a previous malignant cancer during the 2 years preceding the study; investigational study treatment within 28 days or 5 half-lives; pregnancy and/or breast feeding; nausea and vomiting refractory to therapy, malabsorption, external biliary shunt, previous bowel resection; heart attack, unstable angina and/or severe degree, congestive heart failure, cerebrovascular accident or transient ischemic attack, pulmonary embolism, arterial hypertension not adequately controlled in the 6 months before the start of study; history of atrial or ventricular arrhythmia symptomatic > grade 2 (NCI CTCAE); history of retinopathy; uncontrolled glaucoma; serum cholesterol ≥ grade 2; hypertriglyceridemia ≥ grade 2; hyperglycemia (fasting) ≥ grade 2; and correct QT interval > 450 ms to baseline.

From May 2014 to July 2015, enrolled patients have received the BRAF-I vemurafenib 960 mg twice daily (b.i.d.) + PEG-IFN-α-2b 1 µg/kg once weekly, which was started after 15 days of vemurafenib, and have continued until the maximum tolerated dose (MTD) was reached. If the MTD was not reached, PEG-IFN-α-2b dose was escalated to 2 µg/kg, and if MTD was still not reached, then PEG-IFN-α-2b dose was further escalated to 3 µg/kg. In 2015, new evidence has shown that vemurafenib and MEK-I cobimetinib was more effective than vemurafenib alone [22]. Therefore, the protocol was amended, enrollment restarted, and subjects received vemurafenib 960 mg b.i.d. + cobimetinib 60 mg once daily (o.d.) for 21 days/4 weeks + PEG-IFN-α-2b 1 µg/kg once weekly subcutaneously, started after 15 days of vemurafenib + cobimetinib only, and continued until MTD was reached. A cohort of three consecutive patients was treated at each dose level. Patients were scheduled to receive at least two courses of therapy (cycle every 28 days) at the same dose level before escalation.

The dose was reduced as needed in order to maintain an ECOG PS score of 0–1. Treatment was continued until the development of progressive disease (as per investigator assessment), unacceptable toxicity, consent withdrawal, death or up to 24 weeks.

Acetaminophen (500–1000 mg) was given 30 min prior to receiving the first dose of PEG IFN-α-2b; it could be continued as needed and should not exceed 3000 mg/day. All concomitant medication or treatment required by the patient were at the discretion of the treating physician. Other anti-cancer therapies, concomitant alternative therapies and herbal preparations were not allowed during study treatment.

### Outcome measures

Efficacy (RECIST version 1.1) was assessed by Investigators using conventional cross-sectional imaging (CT or MRI).

Adverse events were recorded during the treatment period according to CTCAE v. 4.0.

### Biomarker sample collection and processing

#### Monoclonal antibodies

Monoclonal antibody (mAb) HCA2, which recognizes β2m-free HLA-A (excluding -A24), -B7301 and -G heavy chains [23], and mAb HC10, which recognizes β2m-free HLA-A3, -A10, -A28, -A29, -A30, -A31, -A32, -A33 and all β2m-free -HLA-B (excluding -B5702, -B5804 and -B73) and -HLA-C heavy chains [24, 25], were developed and characterized as previously described. mAbs were purified from ascitic fluid by affinity chromatography on Protein G columns. The purity and specific reactivity of mAb preparations were assessed by SDS-PAGE, binding assays and western blotting, respectively. A pool of mAb HCA2 and HC10 (ratio: 1:1) was used for immunohistochemistry (IHC) of tissues sections.

The IFNAR1- (ab62693, Abcam), CD3- (clone 2GV6, Ventana, Roche), CD8- (clone C8/144b, Dako), and PD-L1- (clone 22C3 pharmDx, Dako) specific mAbs were purchased from the indicated companies.

#### IHC of metastatic melanoma biopsies

Metastatic melanoma biopsies were obtained from the Department of Pathological Anatomy and Cytopathology at Istituto Nazionale Tumori IRCCS–Fondazione “G Pascale”. Presence of tumor cells in formalin-fixed paraffin embedded (FFPE) tissues was monitored by hematoxylin and eosin staining. All tumor samples were reviewed according to the American Joint Committee on Cancer (AJCC) classification criteria, using standard tissue sections and appropriate IHC analyses.

Biomarker expression was investigated on 4 µm-thick FFPE tissue sections obtained from punch biopsies, which were excised no later than 15 days before the start of treatment and after 15 days from the start of therapy.

To assess HLA class I antigen and IFNAR1 expression, tumor tissue sections were subjected to a fully automated staining with mAbs on Bond-III (Leica Biosystem) using the Bond Polymer Refine Detection Kit (DS9800, Leica Biosystem), and counterstained with hematoxylin. IHC staining with CD3- and CD8-specific mAbs was performed according to the manufacturers’ instructions using the autostainer BenchMark XT (Ventana, Roche). IHC staining with PD-L1-specific mAb was performed according to the manufacturer’s instructions using the autostainer Link 48 (Agilent).

HLA class I antigen expression was quantified as a composite score generated by multiplying the staining intensity by the percentage of the stained tumor cells (HLA total score = score intensity × percentage of stained cells). Results were graded as positive, heterogeneous or negative when the HLA total score in an entire lesion was > 100, 25–100 and < 25, respectively [26]. CD3 and CD8 expression was scored by counting the number of lymphocytes on the entire section at 400 × magnification. Membrane PD-L1 expression was evaluated on tumor cells and on tumor-infiltrating lymphocytes being expressed also by lymphocytes, macrophage, dendritic cells, granulocytes [27]. PD-L1 scoring was based on the proportion of tumor cells with membranous expression of PD-L1. Samples with value upper the 1% were considered positive.

IFNAR1 expression was quantified as membrane staining intensity of melanoma cells. Staining intensity was scored as negative (0 +), weak (1 +), moderate (2 +) and strong (3 +). The percentage of stained melanoma cells was calculated over the whole section. All markers were scored by melanoma expert pathologists in a blinded manner.

#### Nanostring gene-expression analysis

RNA from FFPE tumor tissues was extracted using RNeasy FFPE Kit (Qiagen). Purified RNA (100 ng) was used for hybridization and subjected to gene-profiling analysis on NanoString nCounter Sprint Profiler™ through PanCancer IO 360 panel (Nanostring Technologies), characterized by 20 internal references and 770 human genes involved in the interplay between tumor microenvironment and immune response.

Gene data were normalized and analyzed using nSolver Version 4.0 Software; volcano plots were visualized using nSolver Advanced Analysis Version 2.0.115 and R Version 3.3.2 Software (Nanostring Technologies). Due to limited sample sizes, statistical correction for multiple comparisons resulted in a null result for all comparisons. Reported p-values are uncorrected values.

### Statistical analysis

The sample size was based on the classical 3 + 3 design, and a minimum of three patients had to be enrolled. Patients experiencing toxicities that were not dose-limiting were retreated at the same dose level upon full recovery.

Data from this study were tabulated using descriptive statistics. Survival analysis of progression-free survival (PFS; defined as the time from the administration of the first dose of the study drug to documented radiological progression, death or lost to follow-up, whichever occurred first) and overall survival (OS; defined as the time from the administration of the first dose of the study drug to death or lost-to-follow-up, whichever occurred first) was conducted by the Kaplan–Meier method, with an explorative intent, in patients with or without IFNAR1 expression on melanoma cells ≤ 35%.

Statistical evaluations of endpoints were performed using a two-sided significance level of 0.05 unless otherwise specified. Statistical analyses were produced using SAS® for Windows, Version 9.3.

## Results

**Fig. 1:**
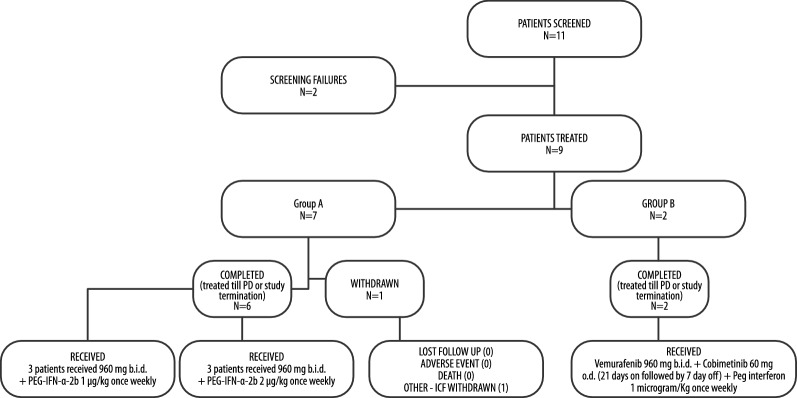
Patient disposition. Group A: patients who received vemurafenib plus PEG-IFN-α-2b, group B: patients who received vemurafenib + cobimetinib + PEG-IFN-α-2b

Overall, 11 patients were screened for enrollment. Two patients were considered as screening failures (one patient had symptomatic brain metastasis and one patient showed abnormal laboratory values), and one patient withdrew his/her consent before receiving the study treatment. Enrolled patients (n = 8; five males) who received treatment were Caucasian, with an age range of 41–78 years (mean: 63.1 years). Two patients had lactate dehydrogenase value > ULN, seven had ECOG PS = 0, and one had ECOG PS = 1. Two patients were in stage M1a, one was in stage M1b, four were in stage M1c and one had unresectable stage IIIb disease. Two patients had asymptomatic brain metastases, and three subjects had ≥ 3 involved organs. Out of eight treated subjects, three patients were treated with vemurafenib 960 mg b.i.d. + PEG-IFN-α-2b 1 µg/kg once weekly and MTD was not reached; three patients were treated with vemurafenib 960 mg b.i.d. + PEG-IFN-α-2b 2 µg/kg once weekly and MTD was not reached. Following the amendment of protocol, two patients were treated with vemurafenib 960 mg b.i.d. + cobimetinib 60 mg o.d. for 21 days/4 weeks + PEG-IFN-α-2b 1 µg/kg and MTD was not reached due to early closure of the clinical trial. During treatment with vemurafenib 960 mg b.i.d. + PEG-IFN-α-2b 1 µg/kg once weekly, dose-limiting toxicity (DLT) neutropenia grade (G)4 and leukopenia G4 related to the study drugs (vemurafenib plus PEG-IFN) has been observed. DLT was not observed in the two patients treated with vemurafenib 960 mg b.i.d. + cobimetinib 60 mg o.d. for 21 days/4 weeks + PEG-IFN-α-2b 1 µg/kg. Patient disposition is shown in Fig. 1.

### Safety

From the beginning of the study until 18 March 2018, 59 adverse events (AEs) were reported; four of them were serious AEs. Three serious AEs were related to study treatments. Two grade 4 AEs, and one grade 3 AE were recorded.

During treatment with vemurafenib 960 mg b.i.d. + PEG-IFN-α-2b 1 µg/kg once weekly, 65.3% of the AEs observed were of mild severity, 28.6% moderate, and 4.1% were life-threatening (neutropenia and leukopenia grade 4). On 1 August 2014, one subject experienced DLT neutropenia G4 and leukopenia G4 related to the study drugs (vemurafenib + Peg-IFN), which completely resolved on 11 August 2014, after treatment with filgrastim. One patient died due to disease progression.

During treatment with vemurafenib 960 mg b.i.d. + PEG-IFN-α-2b 1 µg/kg once weekly + cobimetinib 60 mg o.d., 30% of the EAs observed were mild, 10% moderate and 30% severe. One patient had a temporary interruption of treatment due to a G3 AE, and therapy was restarted with cobimetinib dose reduced to 40 mg to maintain PS.

There were no study drug interruptions due to meaningful changes in vital signs, physical examination, clinical laboratory evaluation and ECG.

### Efficacy

**Fig. 2:**
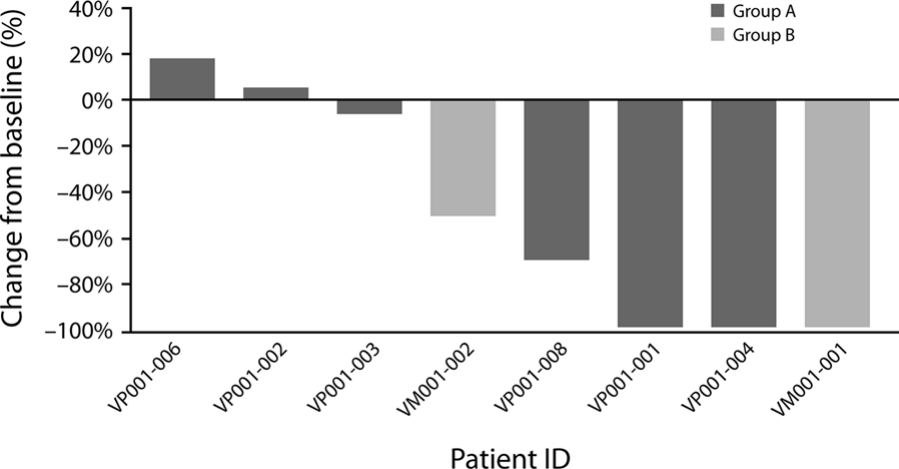
Change in tumor size during the study. Group A: patients who received vemurafenib plus PEG-IFN-α-2b, group B: patients who received vemurafenib + cobimetinib + PEG-IFN-α-2b

**Fig. 3:**
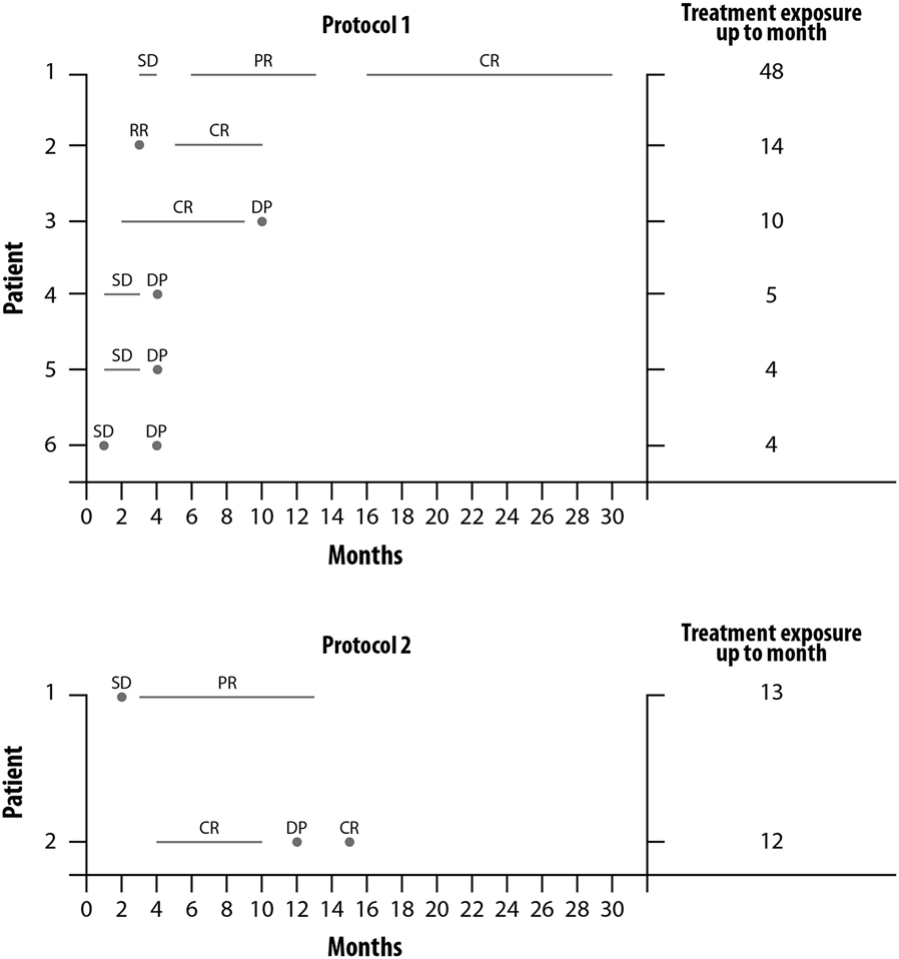
Tumor response during study treatment according to baseline staging. **a** patients who received vemurafenib plus PEG-IFN-α-2b (patients 1 and 3 had durable response); **b** patients who received vemurafenib + cobimetinib + PEG-IFN-α-2b (patient 1 had durable response). CR: complete response, DP: disease progression, PR: partial response, SD: stable disease

During the dose-escalation phase of the study, one (9%) patient achieved complete response, and one (9%) achieved partial response. In both cases, the disease was located on the skin. Both responses continued at the time of data cut-off (March 2018), and durable response rate (at least 32 weeks) was 38%. The mean tumor reduction was − 50.2% (± 50.2%; range: − 100% to − 18%) (Fig. 2). Tumor response during exposure to study treatment is shown in Fig. 3.

Five subjects died during follow-up due to disease progression.

### Association of low IFNAR1 expression level on melanoma cells in pre- and post-treatment biopsies with favorable response to therapy

**Fig. 4:**
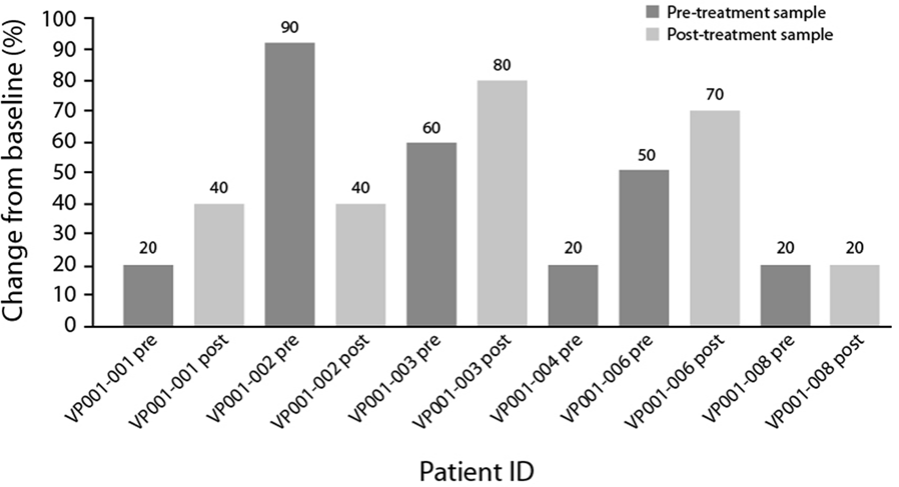
Proportion of IFNAR1-positive cells in tumor samples, in the immunohistochemical analysis

Tissue sections from all patients’ biopsies were examined by hematoxylin and eosin staining. Unfortunately, IHC analysis was performed only on six patients, because in two cases, the tumor component was poorly represented to be examined. For one patient, only the pretreatment specimen was available. The mean percentage of melanoma cells expressing IFNAR1 was 48.0 (± 29.5) in sections obtained before treatment with vemurafenib + PEG-IFN-α-2b, and 46.0 (± 19.5) in sections from post-treatment biopsies (Fig. 4). Although we have observed increased IFNAR1 expression in post-treatment biopsy in three out of five patients, overall, the difference between pre- and post-treatment biopsies was not statistically significant (p = 0.88; paired Student’s t-test), and due to limited tissue availability, we could not assess the activation status of the IFN pathway.

**Fig. 5:**
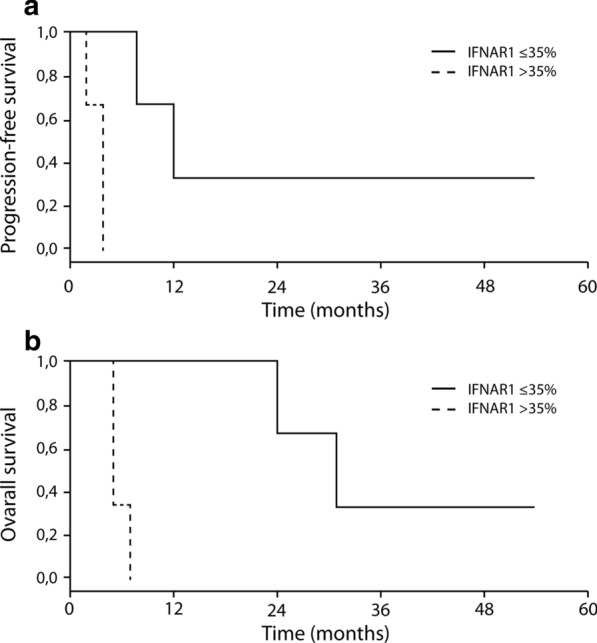
PFS (**a**) and OS (**b**) according to the proportion of IFNAR1-positive cells in pretreatment specimens

Considering as cut-off the median value of melanoma cells expressing IFNAR1 (range: 20–90, Wilcoxon test), patients with a pre-treatment percentage of melanoma cells positive for IFNAR1 of ≤ 35% had a median PFS of 12.0 months (range: 5.6–18.4 months), while patients with a percentage of melanoma cells expressing IFNAR1 of > 35% had a median PFS of 4.0 months (range: 0–8.8 months) (p = 0.03) (Fig. 5a). Regarding OS, patients with a percentage of melanoma cells expressing IFNAR1 of ≤ 35% had a median OS of 31.0 months (range: 19.8–42.2 months), while patients with a percentage of stained melanoma cells expressing IFNAR1 of > 35% had a median OS of 5.0 months (p = 0.02) (Fig. 5b).

Furthermore, even if the data were not statistically supported, we have observed that patients with a low IFNAR1 expression on melanoma cells had a durable complete response to treatment, while patients with a high IFNAR1 expression on melanoma cells had first a stable or partial response, followed by a fast progression disease.

We have evaluated PD-L1 expression in all biopsies and no statistically significant result was found; PD-L1 expression was < 1% in all pre-treatment tissues, only in two post-treatment biopsies PD-L1 expression was > 1%. HLA-A heavy chains were expressed in the membrane of melanoma cells in two biopsies and in the cytoplasm in three biopsies. HLA-B, C heavy chains were detected only in the cytoplasm of melanoma cells in both pre- and post-treatment biopsies. Both HLA-A and HLA-B, C heavy chains had a low expression both in terms of percentage of stained melanoma cells and of staining intensity (see Additional file 1: Table S1). The expression pattern of both of them did not markedly change following treatment. The CD8 and CD3 T-cell infiltration, as determined by IHC staining with monoclonal antibodies of the entire tumor section, was very heterogeneous in terms of both percentage of cells and of staining intensity. No difference between pre- and post-treatment biopsies was detected. HLA-A and HLA-B, C heavy chain expression, as well as lymphocyte infiltration did not correlate with OS, PFS and response to treatment.

### Gene-expression analysis results

**Fig. 6:**
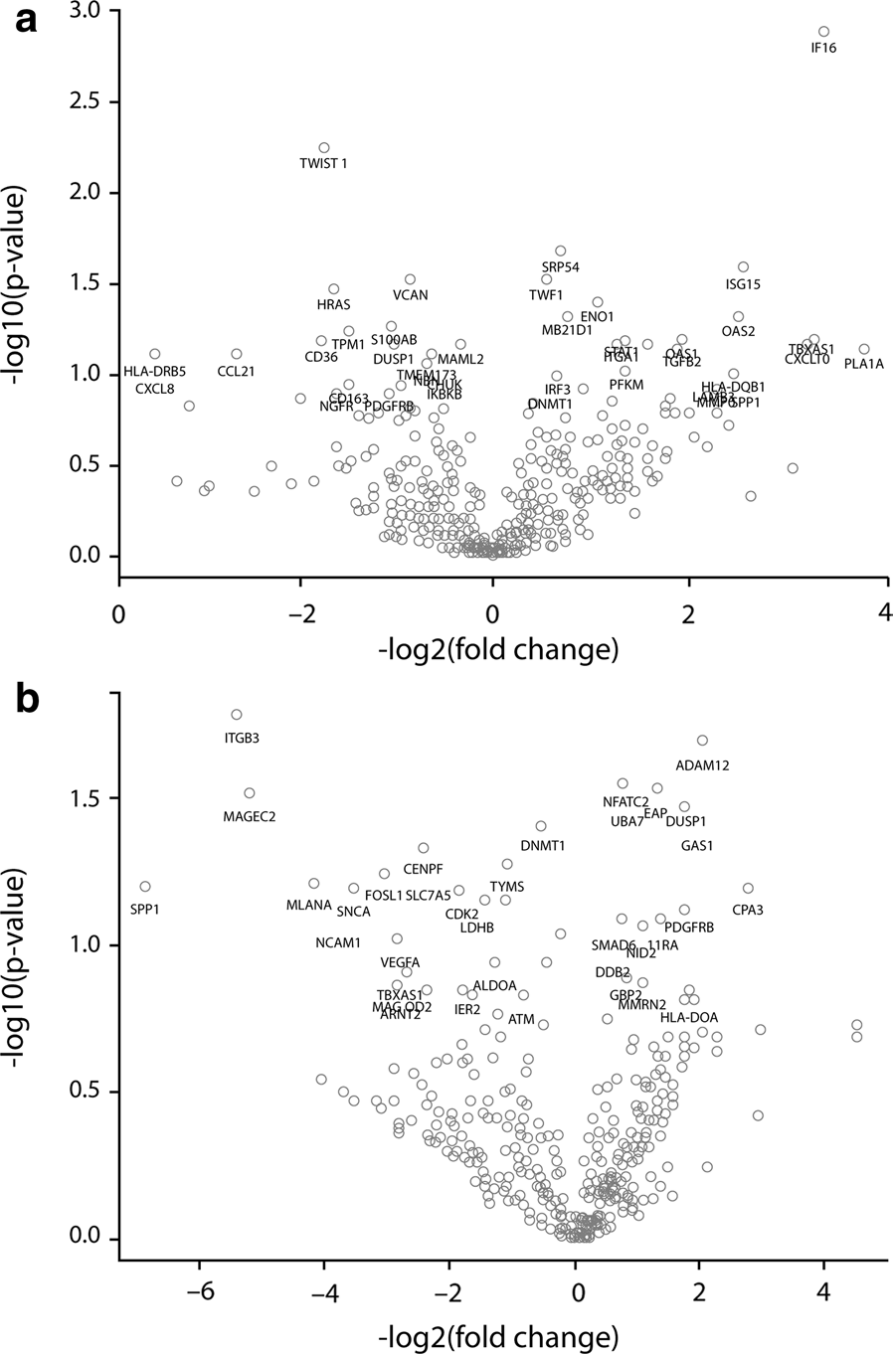
Differential gene expression by Nanostring. **a** Pretreatment: Responders vs. non-responders. **b** Post-treatment vs. pretreatment in responders

In order to further explore the molecular profiles of the patients enrolled in the study, gene-expression analysis was performed on tumor samples from patients. First, we considered differences in baseline gene expression before treatment in responders (OS > 10 weeks) versus non-responders (OS < 10 weeks) (Fig. 6a). Genes involved in the cellular response to IFN, including ISG15 (log2 fold change = 2.56, p = 0.0264) and IFI6 (log2 fold change = 3.37, p = 0.00128), were expressed at higher levels at baseline in responders compared to non-responders. We also considered the effects of treatment within the responder group by taking the differential gene expression between pre-treatment and post-treatment samples (Fig. 6b). Following treatment, responders had higher levels of potent growth suppressors, including *GAS1* (log2 fold change = 2.08; p = 0.0381) and *DUSP1* (log2 fold change = 1.75; p = 0.0323), as well as several genes involved in a metabolically robust immune response, including *FAP* (log2 fold change = 1.32; p = 0.0294). Several cancer biomarker genes were downregulated following treatment in responders, including MLANA (log2 fold change = − 4.19; p = 0.0617) and MAGEC2 (log2 fold change = − 5.19; p = 0.0308).

## Discussion

Vemurafenib is the first BRAF-I developed and approved for the first- and second-line treatment of metastatic melanoma patients harboring the *BRAF*V600 mutation. Treatment with vemurafenib improved OS, PFS and response rate, compared to standard chemotherapy with dacarbazine [28]. Response to vemurafenib treatment results in high rates of initial tumor regression at the PET-CT scan in few weeks and rapid but short-lasting improvement of symptoms. The phase III trial BRIM-3 showed that treatment with vemurafenib resulted in higher PFS compared with standard chemotherapy (5.3 months in the vemurafenib group versus 1.6 months in dacarbazine group) [29]. Targeted therapies in combination with immunological therapies could improve responses and overcome resistance. The first example of this combination was vemurafenib + the CTLA-4-specific mAb, ipilimumab [30], which, however, was considered as not feasible due to an increased hepatotoxicity. The combination of the BRAF-I dabrafenib and the CTLA-4 mAb ipilimumab seemed more feasible. However, the addition of MEK-I to BRAF-I showed an improvement of overall response rate with a delay in the development of resistance, although associated with severe gastrointestinal toxicity [31].

Based on the strong pharmacological rationale of the upregulation of IFNAR1 induced by vemurafenib, we started the phase I VEMPULINT trial, which initially assessed the combination of vemurafenib and PEG-IFN-α-2b, and, after the publication of the evidence that vemurafenib/cobimetinib was more effective than vemurafenib alone [22], assessed the combination of vemurafenib + cobimetinib + PEG-IFN-α-2b. Unfortunately, because of the early interruption of trial, due to the discontinuation of PEG-IFN-α-2b in Europe, no conclusions were given for the recommended dosage.

The AEs observed in our study were mostly mild in severity and were similar in the therapy with and without cobimetinib. The role of the combinations was confirmed by the results of the IHC, showing that inhibition of the MAPK pathway may improve sensibility to IFN-α in melanoma cells. Although assessing efficacy was not the primary aim of this phase I study, only two cases of response were reported, but tumor reduction was observed during the study. Overall, due to the small sample size and the premature termination of the study, no firm conclusions on safety or signs of activity can be drawn. However, we were able to report some intriguing data derived from translational research.

The results of the IHC analysis of the biopsies from the treated patients deserve some comments. Tumor heterogeneity is a significant issue in many tumors, making smaller biopsy samples less reliable for tissue-based biomarkers, such as PD-L1 tumor expression. Despite the small number of analyzed biopsies, we have observed that baseline IFNAR1 membrane expression level correlated with survival and response to treatment. In particular, a low number of melanoma cells expressing IFNAR1 in pretreatment lesions was correlated with a better clinical outcome. It was our intention to also investigate the activation in tumor tissue of the IFN signaling by vemurafenib and vemurafenib + cobimetinib treatment, but due to the limited size of the biopsies, we were unable to do it. Furthermore, we have hypothesized that treatment with BRAF-I would induce an increase in IFNRA1 expression, which in turn would lead to an increase in the sensitivity of melanoma to the antitumor activity of IFN-α. Unfortunately, due to the small number of patients enrolled and biopsies available, although we did observe a trend of increased IFNRA1 expression in some patients after treatment, the data were not statistically significant.

The gene-expression data suggest that patients who will go on to have positive responses to immunotherapy express higher levels of genes related to IFN response. This result agrees with findings by Yan et al. [32]. These authors compared baseline tumor features of melanoma patients who had either complete response or fast progression after treatment with BRAF and MEK inhibitors. Their results suggest that enriched immune infiltration might be a shared feature favoring response to targeted therapy: the analysis of RNA profiles showed that the expression of immune response-related genes was enriched in tumors from patients with complete response [32]. In line with these results, our study suggests that a subset of patients may be particularly primed to benefit from this treatment and could be identified by a prospective molecular screening. Due to the limited number of samples analyzed and the large number of genes tested, none of the differential gene-expression results was statistically significant. However, these data add to our biological understanding of the mechanisms of response to this treatment.

## Conclusion

Our study supports the overall safety of the vemurafenib + PEG-IFN-α-2b + cobimetinib combination. Our main finding was that IFNAR1 expression levels correlated with response to treatment, including survival. Given the data on other combination regimens based on BRAF-I, MEK-I and anti-PD-1 mAb [33, 34], we believe that the combination of vemurafenib, PEG-IFN-α-2b and cobimetinib would have difficulty finding a niche in the current treatment scenario for advanced melanoma. However, we are aware of all the limitations of the study, such as the population and treatment heterogeneity (stage III and stage IV, brain metastases, LDH level, iBRAF in some patients, iBRAF + iMEK in others) and the absence of a control group, but the notion that IFNAR1 levels and expression of genes involved in response to IFN predict treatment response tempts us to speculate that a subgroup of patients may be particularly responsive to the treatment described. This hypothesis needs verification in studies of anti-PD-1/BRAF/MEK combination regimens.

## Supplementary Information

**Additional file 1: Table S1.** HLA-A and HLA-B,C expression on melanoma cells in pre- and post-treatment biopsies. Abbreviation:% = percentage; Int = Intensity; Loc= localization.

## Data Availability

The datasets used and/or analyzed during the current study are available from the corresponding author on reasonable request.

## Abbreviations

AE: Adverse event
BRAF-I: BRAF inhibitor
DLT: Dose-limiting toxicity
ECOG: Eastern Cooperative Oncology Group
FFPE: Formalin-fixed paraffin embedded
G: Grade
IFNAR1: IFN-α receptor-1
IHC: Immunohistochemistry
mAb: Monoclonal antibody
MAPK: MAP kinase
MTD: Maximum tolerated dose
OS: Overall survival
PFS: Progression-free survival
PS: Performance status
