# Supplementary material for: A monocentric phase I study of vemurafenib plus cobimetinib plus PEG-interferon (VEMUPLINT) in advanced melanoma patients harboring the V600BRAF mutation
Source: J Transl Med. 2021 Jan 6;19:17. doi: 10.1186/s12967-020-02680-7 (PMC7789377; doi:10.1186/s12967-020-02680-7)
Supplement: Supplementary file 1 — Additional file 1: Table S1. HLA-A and HLA-B,C expression on melanoma cells in pre- and post-treatment biopsies. Abbreviation:% = percentage; Int = Intensity; Loc= localization. [file 12967_2020_2680_MOESM1_ESM.docx]

| **ID patients** | **HLAI HCA2** | | | | | | |  | **HLAI HC10** | | | | | | |
| --- | --- | --- | --- | --- | --- | --- | --- | --- | --- | --- | --- | --- | --- | --- | --- |
|  | **Pre-treatment** | | |  | **Post-treatment** | | |  | **Pre-treatment** | | |  | **Post-treatment** | | |
|  | **%** | **Int** | **Loc** |  | **%** | **Int** | **Loc** |  | **%** | **Int** | **Loc** |  | **%** | **Int** | **Loc** |
| VP001-001 | 90 | 2 | m |  | 30 | 2 | c |  | 45 | 2 | m |  | 50 | 2 | m |
| VP001-002 | 0 | 0 |  |  | 10 | 1 | c |  | 60 | 1 | c |  | 0 | 0 |  |
| VP001-003 | 10 | 1 | m |  | 50 | 2 | m |  | 40 | 2 | m |  | 60 | 2 | m |
| VP001-006 | 80 | 1 | c |  | 30 | 1 | c |  | 20 | 1 | c |  | 50 | 2 | c |
| VP001-008 | 10 | 1 | c |  | 30 | 1 | c |  | 30 | 1 | c |  | 40 | 1 | c |

Additional file 1: Table S1. HLA-A and HLA-B,C expression on melanoma cells in pre- and post-treatment biopsies. Abbreviation:% = percentage; Int = Intensity; Loc= localization
